# Treatment of Onychomycosis: An Update

**DOI:** 10.4103/0250-474X.49088

**Published:** 2008

**Authors:** A. A. Shirwaikar, T. Thomas, A. Shirwaikar, R. Lobo, K. S. Prabhu

**Affiliations:** Department of Pharmaceutics, Manipal College of Pharmaceutical Sciences, Manipal-576 104, India; 1 Department of Pharmacognosy, Manipal College of Pharmaceutical Sciences, Manipal-576 104, India

**Keywords:** Onchomycosis, nails, antifungal agents, nail lacquers

## Abstract

Fungal infections of skin are one of the most common infections in human beings. The areas which are likely to get infected include the scalp, the hands and the feet. Dermatophytes, yeasts and moulds are the three major fungi responsible for skin infections. Earlier oral antifungal agents were used for treatment of fungal infection in finger and toe nails. The disadvantages of oral antifungal agents are toxicity and longer treatment period. Now medicated nail lacquers have been developed for the treatment of fungal infections i.e. onychomycosis, which has less toxicity and shorter treatment period.

Finger nails and toe nails are made up of protein and are a form of modified hair. The nail is composed of elements such as, nail matrix or the nail root which is the growing part, under the skin under the nail proximal end. Eponychium or cuticle is the fold of the skin at the proximal end of the skin and paronychium which is the fold of skin on the side of the nail. Hyponychium is the attachments between the skin of the finger or toe and the distal end of the nail. Nail plate, the hard and the translucent portion is composed of keratin and adherent connective tissue that underlies the nail is the nail bed. Lunula, which is the crescent shaped whitish area of the nail bed^1^.

Nail grows at an average rate of 0.1 mm/day (1 cm every 100 days). Finger nails require 3 to 6 months to re-grow completely while toe nails require 12 to 18 months. The actual growth rate depends upon the age, season, exercise level and hereditary factor. The growth record of the nail can show the history of recent health and physiological imbalances and has been used as a diagnostic tool earlier. Major illness will cause a deep horizontal groove on the nails, discoloration, thinning, thickening, brittleness, splitting, grooves, mee's lines, small white lines, small white spots, receded lunula, clubbing (convex) all of which indicate illness in other part of the body. Nails can also be thickened (onychogryphosis), loosened (onycholysis), infected with fungus (onychomycosis) or degenerate (onychodystrophy).

## ONYCHOMYCOSIS

Onychomycosis is an infection of the nail caused by fungi such as dermatophytes, non-dermatophyte moulds and yeasts (mainly *Candida* species). Of these 80% of the toenail infections are caused by dermatophytes (*Trichophyton rubrum*). Onychomycosis is classified clinically as distal and lateral subungual onychomycosis (DLSO), superficial white onychomycosis (SWO), proximal subungual onychomycosis (PSO), candidial onychomycosis and total dystrophic onychomycosis.

### Distal and lateral subungual onychomycosis:

Distal and lateral subungual onychomycosis are seen in majority of cases and is almost always due to dermatophyte infection. It affects the hyponychium, often at the lateral edges initially, and spreads proximally along the nail bed resulting in subungual hyperkeratosis and onycholysis although the nail plate is not initially affected. Distal and lateral subungual onychomycosis may be confined to one side of the nail or spread sideways to involve the whole of the nail bed, and progresses relentlessly until it reaches the posterior nail fold. Eventually the nail plate becomes friable and may break up, often due to trauma, although nail destruction may be related to invasion of the plate by dermatophytes that have keratolytic properties. Examination of the surrounding skin will nearly always reveal evidence of tinea pedis. Toenail infection is an almost inevitable precursor of fingernail dermatophytosis, which has a similar clinical appearance although nail thickening is not as common^2^.

### Superficial white onychomycosis:

Superficial white onychomycosis is also, also a dermatophyte infection, which is caused by *T. mentagrophytes*. It is much less common than distal and lateral subungual onychomycosis and affects the surface of the nail plate rather than the nail bed. Discoloration is white rather than cream and the surface of the nail plate is noticeably flaky. Onycholysis is not a common feature of superficial white onychomycosis and intercurrent foot infection is not as frequent as in distal and lateral subungual onychomycosis.

### Proximal subungual onychomycosis:

Proximal subungual onychomycosis is an uncommon variety of dermatophyte infection often related to intercurrent disease. Immunosuppressed patients, notably those who are human immunodeficiency virus-positive, may have a variety of dermatophyte infection; conditions such as peripheral vascular disease and diabetes also may present in this way. Evidence of intercurrent disease should therefore be considered in a patient with proximal subungual onychomycosis.

### Candidal onychomycosis:

Infection of the nail with *Candida* yeasts may present in one of the following four ways, (i) chronic paronychia with secondary nail dystrophy; (ii) distal nail infection; (iii) chronic mucocutaneous candidiasis; and (iv) secondary candidiasis.

Chronic paronychia of the fingernails generally occurs in patients with wet occupations. Swelling of the posterior nail fold occurs secondary to chronic immersion in water or possibly due to allergic reactions to some foods, and the cuticle becomes detached from the nail plate thus losing its water-tight properties. Microorganisms, both yeasts and bacteria, enter the subcuticular space causing further swelling of the posterior nail fold. Distal nail infection with *Candida* yeasts is uncommon and virtually all patients have Raynaud's phenomenon or some other form of vascular insufficiency.

Chronic mucocutaneous candidiasis, involves the mucous membranes which is caused due to diminished cell-mediated immunity. Clinical signs vary with the severity of immunosuppression, but in more severe cases gross thickening of the nails occurs, amounting to a *Candida* granuloma. Secondary candidal onychomycosis occurs due to other diseases of the nail, mostly psoriasis^2^.

## DIAGNOSIS

Fifty percent of all nail dystrophy are fungal in origin; it is not always possible to identify such cases accurately. Treatment period of the nail are mostly long-term and it takes time for the nail to grow completely before the treatment can be rendered as successful. Laboratory diagnosis consists of microscopy to visualize fungal elements in the nail sample and culture to identify the species concerned. The success of such tests depends upon the quality of the sample, the experience of the microbiologist and the ability of the laboratory to discriminate between organisms that are likely pathogens, organisms growing in the nail as saprophytes, and contamination of the culture plate. The addition of Parker's blue or black ink may enhance visualization of the sample^3^.

### Why is the treatment necessary?

Onychomycosis, leads to discoloration and deformation of the nails. Particular problems include thickening, which may cause pain and make basic nail cutting difficult. In patients with complicating factors, deformed nails can lead to surrounding tissue damage and once again promote secondary bacterial infection. Furthermore, a recent study has highlighted the psychological, social and occupational effects of the condition which appear to have been underestimated by health care professionals for treating the condition.

## TREATMENT

Both topical and oral agents are available for the treatment of fungal nail infection. The primary aim of treatment is to eradicate the organism as demonstrated by microscopy and culture. This is defined as the primary end-point in almost all properly conducted studies. Clinical improvement and clinical cure are secondary end-points based on a strict scoring system of clinical abnormalities in the nail apparatus. It must be recognized that successful eradication of the fungus does not always render the nails normal as they may have been dystrophic prior to infection. Such dystrophy may be due to trauma or non fungal nail disease.

### Oral therapy:

The most commonly used oral drugs for treatment of onychomycosis is griseofulvin, terbinafine, itraconazole and ketoconazole. The disadvantages of oral antifungal agents are, they require a longer treatment period and they have more side effects, e.g. terbinafine (Lamisil®). This drug is taken daily for 8 weeks for fingernail fungus and for 12 weeks for toenail fungus. The most frequent side effects of Lamisil® are headache, gastrointestinal disturbance (diarrhea and/or dyspepsia), rash and elevated liver enzymes^4^.

Itraconazole (Sporanox®) this is often prescribed in “pulse doses” one week per month for 2 or 3 months. It can interact with some commonly used drugs such as the antibiotic erythromycin or certain asthma medications. The most frequent side effects of Sporanox® include increased liver function tests, skin rash, high triglycerides, and gastrointestinal effects (nausea, bloating, and diarrhea)^4^. Ketoconazole (Diflucan®) may be given once a week for several months. The most common side effects are headache, skin rash, and/or gastrointestinal (GI) disturbance (nausea, vomiting, diarrhea, and/or abdominal pain)^4^.

Griseofulvin (Fulvicin®, Gifulvin®, Gris-Peg®) this drug has been the main stay of oral antifungal therapy for many years. Although this drug is safe, it is not very effective against toenail fungus^4^.

### Topical Therapy:

Creams and other topical medications are usually not effective against nail fungus. This is because nails are too hard for external applications to penetrate. However, a new medicated nail lacquer has been approved to treat finger or toenail fungus that does not involve the white portion of the nail (lunula) in persons with normal immune systems.

The nail lacquers which are currently available are ciclopirox and amorolfine nail lacquers, which are effective for the treatment or prevention of fungal infections such as onychomycosis. The nail lacquer consists of fungicidally effective amount of ciclopirox, amorolfine, or other antifungal agent in a clear, stable, film-forming lacquer vehicle; a water-insoluble film-forming polymer; 2-*n*-nonyl-1,3-dioxolane or similar penetration enhancer; and volatile solvent. A plasticizer is used for the film-forming polymer which is also compatible with the other components and the preferred penetration enhancers may also function as plasticizer. The composition, when applied to the nails provides a hard, clear, water-resistant film containing the antifungal agent. The film is resistant to multiple washings and is effective in the treatment of onychomycosis^4^.

### Ciclopirox (Penlac 8%) Nail Lacquers:

Ciclopirox targets a variety of metabolic processes in the fungal cell. It chelates with the polyvalent cations (Fe^3+^ and Al^3+^) that are involved in fungal enzymatic activity, ultimately interrupting intracellular energy production and toxic peroxide degradation. Ciclopirox may also inhibit fungal nutrient uptake, resulting in depletion of amino acids and nucleotides and reduction in protein synthesis.

The most common are rash-related adverse effects such as periungual erythema and erythema of the proximal nail fold were reported more frequently in patients treated with ciclopirox nail lacquer topical solution, i.e., 8%. Other adverse effects which were thought to be causally related included nail disorders such as shape change, irritation, ingrown toenail, and discoloration^5^.

### Amorolfine (5%) Nail Lacquers:

Amorolfine is a topical antimycotic (antifungal) agent that has a fungicidal effect. It inhibits sterol biosynthesis and thereby disrupts the fungal cell membrane leading to cell death. Amorolfine is a broad spectrum antimycotic with activity against a wide range of organisms including dermatophytes (*Trichophyton, Microsporum* and *Epidermophyton* species), yeasts (*Candida, Cryptococcus* and *Malassezia* species) and moulds. When the amorolfine nail lacquer is applied to the nail surface the solvent evaporates to leave a highly concentrated deposit of amorolfine in an occlusive film on the nail. Now this acts as depot from which amorolfine penetrates and diffuses through the nail plate over the next seven days. In this way, amorolfine is delivered to the nail bed. Amorolfine has to be applied regularly until all the affected nail tissue has grown out. This takes 9 to 12 months for toenails and six months for fingernails^6^.

With the use of topical Amorolfine cream (0.125% to 0.5%) in superficial fungal infections, local adverse reactions have occurred in 2% to 7% of patients. Itching, burning, erythema and scaling have been reported most frequently. Other effects include irritation, exudation, blistering, inflammation, edema, eczematous reaction and dermatitis.

In patients with onychomycosis treated with 5% amorolfine lacquer, the incidence of local adverse effects has been low (1% or less). Local effects like burning, pruritus, vesicles, pain or stinging around the nail bed have been reported although a causal relationship was not established^7^. Systemic adverse effects have not been reported with topical use of amorolfine cream or nail lacquer in available studies.

### Precautions:

If a reaction suggesting sensitivity or chemical irritation occurs with the use of Ciclopirox nail lacquer (8%) or Amorolfine nail lacquer (5%) treatment should be discontinued and appropriate therapy suggested. So far there is no relevant clinical experience with patients with insulin dependent diabetes or who have diabetic neuropathy. The risk of removal of the unattached, infected nail, by the health care professional and trimming by the patient, should be carefully considered before prescribing to patients with a history of insulin dependent diabetes mellitus or diabetic neuropathy^8^.

### Information for patients:

Patients should have detailed instructions regarding the use of Penlac nail lacquer (Ciclopirox) topical solution (8%) or Amorolfine nail lacquer (5%) for onychomycosis. The patient should be told to use Penlac nail lacquer (8%) or Amorolfine nail lacquer (5%), as directed by a health care professional. Avoid contact with the eyes and mucous membranes. Contact with skin other than skin immediately surrounding the treated nails should be avoided. Penlac nail lacquer (Ciclopirox) or Amorolfine nail lacquer (5%) is for external use only^9^.

Penlac nail lacquer (Ciclopirox) or Amorolfine nail lacquer (5%) should be applied evenly over the entire nail plate and 5 mm of surrounding skin. If possible, Penlac nail lacquer (ciclopirox) (8%) or Amorolfine nail lacquer (5%) should be applied to the nail bed, hyponychium, and the under surface of the nail plate when it is free of the nail bed (e.g. onycholysis). Contact with the surrounding skin may produce mild, transient irritation (redness).

Removal of the unattached, infected nail, as frequently as monthly, by a health care professional is needed with use of this medication. Inform a health care professional if they have diabetes or problems with numbness in your toes or fingers for consideration of the appropriate nail management program^10^.

Inform a health care professional if the area of application shows signs of increased irritation (redness, itching, burning, blistering, swelling, oozing). Up to 48 weeks of daily applications with Penlac nail lacquer (8%) or Amorolfine nail lacquer (5%) and professional removal of the unattached, infected nail, as frequently as monthly, are considered the full treatment needed to achieve a clear or almost clear nail (defined as 10% or less residual nail involvement). Six months of therapy with professional removal of the unattached, infected nail may be required before initial improvement of symptoms is noticed.

A completely clear nail may not be achieved with the use of this medication. In clinical studies less than 12% of patients were able to achieve either a completely clear or almost clear toenail. Do not use the medication for any disorder other than that for which it is prescribed. Do not use nail polish or other nail cosmetic products on the treated nails. Avoid use near heat or open flame, because product is flammable.

## CONCLUSION

Now-a-days lot of studies are going on with different drugs for use in medicated nail lacquers for the treatment of onychomycosis. The day is not far when onychomycosis will be treated with externally applied drugs only.
